# (*E*)-*N*′-(4-Chloro­benzyl­idene)-1-benzofuran-2-carbohydrazide monohydrate

**DOI:** 10.1107/S1600536812027523

**Published:** 2012-06-23

**Authors:** Hoong-Kun Fun, Ching Kheng Quah, Nitinchandra, Balakrishna Kalluraya, M. Babu

**Affiliations:** a X-ray Crystallography Unit, School of Physics, Universiti Sains Malaysia, 11800 USM, Penang, Malaysia; b Department of Studies in Chemistry, Mangalore University, Mangalagangotri, Mangalore 574 199, India

## Abstract

The title compound, C_16_H_11_ClN_2_O_2_·H_2_O, exists in an *E* conformation with respect to the N=C bond. The benzofuran ring system forms a dihedral angle of 1.26 (4)° with the benzene ring. In the crystal, mol­ecules are linked *via* (N,C)—H⋯O bifurcated acceptor hydrogen bonds and (O,O,C)—H⋯O trifurcated acceptor hydrogen bonds, forming layers parallel to the *bc* plane.

## Related literature

For general background to hydrazone derivatives, see: Sridhar & Perumal (2003 ▶); Vijayakumar *et al.* (2011 ▶). For standard bond-length data, see: Allen *et al.* (1987 ▶). For the stability of the temperature controller used in the data collection, see: Cosier & Glazer (1986 ▶). For related structures, see: Fun, Quah & Abdel-Aziz (2012 ▶); Fun, Quah, Nitinchandra *et al.* (2012 ▶); Fun, Quah, Shyma *et al.* (2012 ▶).
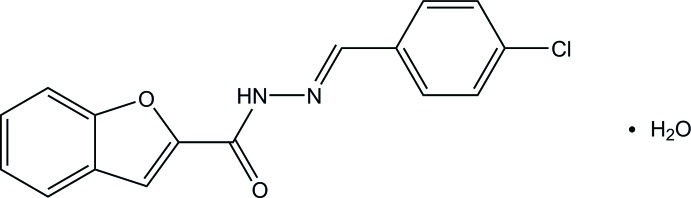


## Experimental

### 

#### Crystal data

C_16_H_11_ClN_2_O_2_·H_2_O
*M* *_r_* = 316.73
Monoclinic, 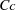

*a* = 24.6121 (15) Å
*b* = 4.6625 (3) Å
*c* = 12.6570 (8) Å
β = 99.294 (1)°
*V* = 1433.37 (16) Å^3^
*Z* = 4
Mo *K*α radiation
μ = 0.28 mm^−1^
*T* = 100 K
0.57 × 0.34 × 0.09 mm

#### Data collection

Bruker SMART APEXII DUO CCD area-detector diffractometer
Absorption correction: multi-scan (*SADABS*; Bruker, 2009 ▶) *T*_min_ = 0.856, *T*_max_ = 0.975
7338 measured reflections
4620 independent reflections
4511 reflections with *I* > 2σ(*I*)
*R*_int_ = 0.019

#### Refinement

*R*[*F* ^2^ > 2σ(*F* ^2^)] = 0.028
*wR*(*F* ^2^) = 0.073
*S* = 1.04
4620 reflections
211 parameters
2 restraints
H atoms treated by a mixture of independent and constrained refinement
Δρ_max_ = 0.34 e Å^−3^
Δρ_min_ = −0.19 e Å^−3^
Absolute structure: Flack (1983 ▶), 2025 Friedel pairs
Flack parameter: 0.03 (3)

### 

Data collection: *APEX2* (Bruker, 2009 ▶); cell refinement: *SAINT* (Bruker, 2009 ▶); data reduction: *SAINT*; program(s) used to solve structure: *SHELXTL* (Sheldrick, 2008 ▶); program(s) used to refine structure: *SHELXTL*; molecular graphics: *SHELXTL*; software used to prepare material for publication: *SHELXTL* and *PLATON* (Spek, 2009 ▶).

## Supplementary Material

Crystal structure: contains datablock(s) global, I. DOI: 10.1107/S1600536812027523/is5157sup1.cif

Structure factors: contains datablock(s) I. DOI: 10.1107/S1600536812027523/is5157Isup2.hkl

Supplementary material file. DOI: 10.1107/S1600536812027523/is5157Isup3.cml

Additional supplementary materials:  crystallographic information; 3D view; checkCIF report

## Figures and Tables

**Table 1:** Hydrogen-bond geometry (Å, °)

*D*—H⋯*A*	*D*—H	H⋯*A*	*D*⋯*A*	*D*—H⋯*A*
O1*W*—H1*W*1⋯O2	0.855 (19)	2.040 (19)	2.8815 (11)	168.1 (18)
O1*W*—H2*W*1⋯O2^i^	0.75 (2)	2.06 (2)	2.8045 (11)	173 (2)
N1—H1*N*1⋯O1*W* ^ii^	0.909 (19)	1.952 (19)	2.8083 (12)	156.2 (18)
C2—H2*A*⋯O2^ii^	0.95	2.57	3.3710 (14)	142
C10—H10*A*⋯O1*W* ^ii^	0.95	2.54	3.3067 (13)	138

Symmetry codes: (i) 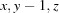; (ii) 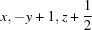.

**Fig. 1.**
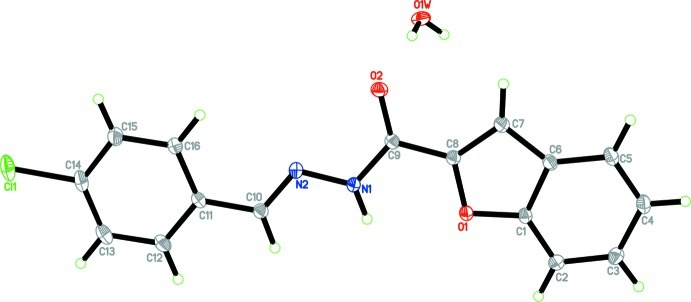
The molecular structure of the title compound showing 50% probability displacement ellipsoids for non-H atoms.

**Fig. 2.**
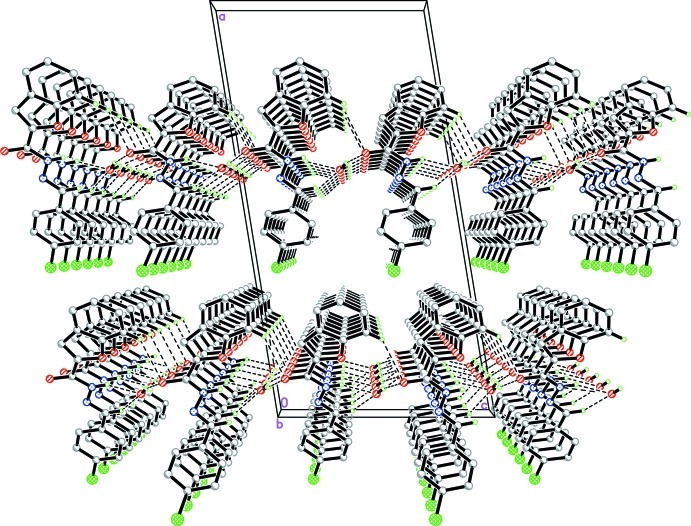
The crystal structure of the title compound, viewed along the b axis. H atoms not involved in hydrogen bonds (dashed lines) have been omitted for clarity.

## supplementary crystallographic information

### Comment

Hydrazones are versatile intermediates and important building blocks. Aryl hydrazones are important building blocks for the synthesis of a variety of heterocyclic compounds such as pyrazolines and pyrazoles (Sridhar & Perumal, 2003). Hydrazones of aliphatic and aromatic methyl ketones yield pyrazole-4-carboxaldehyde on formylation by treatment with Vilsmeier reagent. Hydrazones derived from anisaldehyde and 4-nitro-5-ethoxycarbonyl phenylhydrazine showed excellent NLO property (Vijayakumar *et al.*, 2011). Prompted by these observations, the title compound was synthesized and its crystal structure is reported.

The title compound (Fig. 1) consists of a *N*'-[4-chlorophenyl)methylidene]-1-benzofuran-2-carbohydrazide molecule and a water molecule in the asymmetric unit and exists in an *E* configuration with respect to the N2═C10 bond [1.2848 (13) Å]. The benzofuran ring system (O1/C1-C8, r.m.s deviation = 0.012 Å) forms a dihedral angle of 1.26 (4)° with the benzene ring (C11–C16). Bond lengths (Allen *et al.*, 1987) and angles are within normal ranges and are comparable to related structures (Fun, Quah & Abdel-Aziz, 2012; Fun, Quah, Nitinchandra *et al.*, 2012; Fun, Quah, Shyma *et al.*, 2012).

In the crystal (Fig. 2), molecules are linked *via* intermolecular N1—H1N1···O1W, C10—H10A···O1W bifurcated acceptor hydrogen bonds and O1W—H2W1···O2, O1W—H2W1···O2, C2—H2A···O2 trifurcated acceptor hydrogen bonds (Table 1) to form two-dimensional layers parallel to (100).

### Experimental

The title compound was obtained by refluxing a mixture of 1-benzofuran-2-carbohydrazide (0.01 mol), 4-chlorobenzaldehyde (0.01 mol) in ethanol (30 ml) and 3 drops of concentrated sulfuric acid for 1 h. Excess ethanol was removed from the reaction mixture under reduced pressure. The solid product obtained was filtered, washed with ethanol and dried. Single crystals suitable for X-ray analysis were obtained by slow evaporation of an ethanol-*N*,*N*-dimethylformamide (DMF) (3:1) solution.

### Refinement

N-bound and O-bound H atoms were located in a difference Fourier map and refined freely [N—H = 0.909 (18) Å, and O—H = 0.75 (3) and 0.857 (19) Å]. The rest of hydrogen atoms were positioned geometrically and refined using a riding model with C—H = 0.95 Å and *U*_iso_(H) = 1.2*U*_eq_(C).

### Crystal data

**Table:**

C_16_H_11_ClN_2_O_2_·H_2_O	*F*(000) = 656
*M**_r_* = 316.73	*D*_x_ = 1.468 Mg m^−^^3^
Monoclinic, *C**c*	Mo *K*α radiation, λ = 0.71073 Å
Hall symbol: C -2yc	Cell parameters from 5261 reflections
*a* = 24.6121 (15) Å	θ = 3.3–32.6°
*b* = 4.6625 (3) Å	µ = 0.28 mm^−^^1^
*c* = 12.6570 (8) Å	*T* = 100 K
β = 99.294 (1)°	Plate, yellow
*V* = 1433.37 (16) Å^3^	0.57 × 0.34 × 0.09 mm
*Z* = 4	

### Data collection

**Table:**

Bruker SMART APEXII DUO CCD area-detector diffractometer	4620 independent reflections
Radiation source: fine-focus sealed tube	4511 reflections with *I* > 2σ(*I*)
Graphite monochromator	*R*_int_ = 0.019
φ and ω scans	θ_max_ = 32.6°, θ_min_ = 1.7°
Absorption correction: multi-scan (*SADABS*; Bruker, 2009)	*h* = −35→36
*T*_min_ = 0.856, *T*_max_ = 0.975	*k* = −6→7
7338 measured reflections	*l* = −19→18

### Refinement

**Table:**

Refinement on *F*^2^	Secondary atom site location: difference Fourier map
Least-squares matrix: full	Hydrogen site location: inferred from neighbouring sites
*R*[*F*^2^ > 2σ(*F*^2^)] = 0.028	H atoms treated by a mixture of independent and constrained refinement
*wR*(*F*^2^) = 0.073	*w* = 1/[σ^2^(*F*_o_^2^) + (0.0441*P*)^2^ + 0.2352*P*] where *P* = (*F*_o_^2^ + 2*F*_c_^2^)/3
*S* = 1.04	(Δ/σ)_max_ = 0.002
4620 reflections	Δρ_max_ = 0.34 e Å^−^^3^
211 parameters	Δρ_min_ = −0.19 e Å^−^^3^
2 restraints	Absolute structure: Flack (1983), 2025 Friedel pairs
Primary atom site location: structure-invariant direct methods	Flack parameter: 0.03 (3)

### Special details

**Table:**

Experimental. The crystal was placed in the cold stream of an Oxford Cryosystems Cobra open-flow nitrogen cryostat (Cosier & Glazer, 1986) operating at 100.0 (1) K.
Geometry. All esds (except the esd in the dihedral angle between two l.s. planes) are estimated using the full covariance matrix. The cell esds are taken into account individually in the estimation of esds in distances, angles and torsion angles; correlations between esds in cell parameters are only used when they are defined by crystal symmetry. An approximate (isotropic) treatment of cell esds is used for estimating esds involving l.s. planes.
Refinement. Refinement of F^2^ against ALL reflections. The weighted R-factor wR and goodness of fit S are based on F^2^, conventional R-factors R are based on F, with F set to zero for negative F^2^. The threshold expression of F^2^ > 2sigma(F^2^) is used only for calculating R-factors(gt) etc. and is not relevant to the choice of reflections for refinement. R-factors based on F^2^ are statistically about twice as large as those based on F, and R- factors based on ALL data will be even larger.

### Fractional atomic coordinates and isotropic or equivalent isotropic displacement parameters (Å^2^)

**Table:**

	*x*	*y*	*z*	*U*_iso_*/*U*_eq_	
Cl1	0.362378 (12)	2.08150 (5)	0.62418 (2)	0.02405 (7)	
O1	0.66851 (3)	0.45040 (16)	0.85030 (6)	0.01364 (14)	
O2	0.61809 (3)	0.76090 (16)	0.59506 (6)	0.01549 (14)	
N1	0.59805 (4)	0.87204 (18)	0.76135 (7)	0.01207 (15)	
N2	0.56127 (4)	1.07881 (18)	0.71696 (7)	0.01267 (15)	
C1	0.70999 (4)	0.2512 (2)	0.87293 (8)	0.01263 (16)	
C2	0.72801 (5)	0.1294 (2)	0.97208 (8)	0.01605 (18)	
H2A	0.7119	0.1758	1.0332	0.019*	
C3	0.77114 (5)	−0.0650 (2)	0.97685 (9)	0.0174 (2)	
H3A	0.7851	−0.1537	1.0433	0.021*	
C4	0.79475 (5)	−0.1341 (2)	0.88576 (9)	0.01846 (19)	
H4A	0.8245	−0.2663	0.8922	0.022*	
C5	0.77532 (5)	−0.0121 (2)	0.78699 (9)	0.01828 (19)	
H5A	0.7911	−0.0607	0.7256	0.022*	
C6	0.73181 (4)	0.1849 (2)	0.77992 (8)	0.01348 (17)	
C7	0.70075 (4)	0.3521 (2)	0.69570 (8)	0.01474 (17)	
H7A	0.7049	0.3542	0.6224	0.018*	
C8	0.66431 (4)	0.5062 (2)	0.74233 (8)	0.01243 (16)	
C9	0.62453 (4)	0.7225 (2)	0.69371 (8)	0.01223 (17)	
C10	0.53404 (4)	1.2045 (2)	0.78210 (8)	0.01311 (17)	
H10A	0.5403	1.1551	0.8559	0.016*	
C11	0.49318 (4)	1.4248 (2)	0.74216 (8)	0.01304 (17)	
C12	0.46196 (5)	1.5490 (2)	0.81314 (9)	0.01721 (19)	
H12A	0.4686	1.4950	0.8865	0.021*	
C13	0.42134 (5)	1.7509 (2)	0.77787 (10)	0.0192 (2)	
H13A	0.3998	1.8321	0.8261	0.023*	
C14	0.41298 (4)	1.8308 (2)	0.67104 (10)	0.01754 (19)	
C15	0.44432 (5)	1.7160 (2)	0.59957 (9)	0.0181 (2)	
H15A	0.4386	1.7771	0.5270	0.022*	
C16	0.48413 (4)	1.5106 (2)	0.63496 (8)	0.01574 (18)	
H16A	0.5052	1.4284	0.5861	0.019*	
O1W	0.58492 (4)	0.25083 (19)	0.47297 (6)	0.01780 (15)	
H1W1	0.5989 (8)	0.402 (4)	0.5046 (15)	0.026 (5)*	
H2W1	0.5934 (10)	0.127 (5)	0.510 (2)	0.044 (6)*	
H1N1	0.6041 (8)	0.820 (4)	0.8315 (15)	0.021 (4)*	

### Atomic displacement parameters (Å^2^)

**Table:**

	*U*^11^	*U*^22^	*U*^33^	*U*^12^	*U*^13^	*U*^23^
Cl1	0.01451 (11)	0.01539 (10)	0.03983 (16)	0.00472 (8)	−0.00296 (10)	−0.00640 (11)
O1	0.0150 (3)	0.0136 (3)	0.0126 (3)	0.0039 (2)	0.0033 (3)	0.0011 (2)
O2	0.0206 (4)	0.0144 (3)	0.0122 (3)	0.0019 (3)	0.0046 (3)	0.0009 (3)
N1	0.0125 (4)	0.0121 (3)	0.0117 (3)	0.0020 (3)	0.0019 (3)	0.0011 (3)
N2	0.0120 (4)	0.0116 (3)	0.0144 (4)	0.0009 (3)	0.0018 (3)	0.0008 (3)
C1	0.0126 (4)	0.0109 (4)	0.0144 (4)	0.0010 (3)	0.0024 (3)	−0.0005 (3)
C2	0.0175 (5)	0.0169 (4)	0.0137 (4)	0.0026 (4)	0.0024 (4)	0.0009 (3)
C3	0.0179 (5)	0.0170 (4)	0.0164 (5)	0.0023 (3)	0.0004 (4)	0.0019 (3)
C4	0.0165 (5)	0.0183 (4)	0.0207 (5)	0.0060 (4)	0.0034 (4)	0.0012 (4)
C5	0.0186 (5)	0.0187 (4)	0.0185 (5)	0.0062 (4)	0.0060 (4)	−0.0003 (4)
C6	0.0140 (4)	0.0130 (4)	0.0140 (4)	0.0017 (3)	0.0040 (3)	0.0003 (3)
C7	0.0165 (4)	0.0147 (4)	0.0135 (4)	0.0023 (3)	0.0038 (3)	0.0009 (3)
C8	0.0134 (4)	0.0120 (4)	0.0121 (4)	0.0012 (3)	0.0026 (3)	0.0009 (3)
C9	0.0134 (4)	0.0106 (4)	0.0131 (4)	−0.0003 (3)	0.0034 (3)	0.0003 (3)
C10	0.0140 (4)	0.0127 (4)	0.0129 (4)	0.0007 (3)	0.0029 (3)	0.0007 (3)
C11	0.0124 (4)	0.0122 (4)	0.0150 (4)	0.0002 (3)	0.0039 (3)	−0.0022 (3)
C12	0.0193 (5)	0.0159 (4)	0.0180 (4)	0.0011 (4)	0.0077 (4)	−0.0020 (4)
C13	0.0169 (5)	0.0166 (4)	0.0257 (5)	0.0017 (4)	0.0080 (4)	−0.0050 (4)
C14	0.0116 (4)	0.0119 (4)	0.0284 (5)	0.0014 (3)	0.0009 (4)	−0.0040 (4)
C15	0.0175 (5)	0.0170 (4)	0.0187 (5)	0.0039 (3)	0.0000 (4)	−0.0012 (4)
C16	0.0153 (4)	0.0166 (4)	0.0155 (4)	0.0044 (3)	0.0028 (3)	−0.0007 (3)
O1W	0.0256 (4)	0.0159 (3)	0.0116 (3)	−0.0015 (3)	0.0022 (3)	0.0006 (3)

### Geometric parameters (Å, º)

**Table:**

Cl1—C14	1.7407 (11)	C6—C7	1.4371 (14)
O1—C1	1.3757 (12)	C7—C8	1.3566 (14)
O1—C8	1.3785 (12)	C7—H7A	0.9500
O2—C9	1.2459 (12)	C8—C9	1.4696 (13)
N1—C9	1.3505 (12)	C10—C11	1.4690 (14)
N1—N2	1.3785 (12)	C10—H10A	0.9500
N1—H1N1	0.909 (18)	C11—C16	1.3975 (14)
N2—C10	1.2848 (13)	C11—C12	1.3984 (14)
C1—C2	1.3837 (14)	C12—C13	1.3931 (16)
C1—C6	1.4049 (13)	C12—H12A	0.9500
C2—C3	1.3896 (15)	C13—C14	1.3856 (18)
C2—H2A	0.9500	C13—H13A	0.9500
C3—C4	1.4097 (16)	C14—C15	1.3876 (16)
C3—H3A	0.9500	C15—C16	1.3916 (15)
C4—C5	1.3863 (16)	C15—H15A	0.9500
C4—H4A	0.9500	C16—H16A	0.9500
C5—C6	1.4025 (14)	O1W—H1W1	0.857 (19)
C5—H5A	0.9500	O1W—H2W1	0.75 (3)
			
C1—O1—C8	105.55 (8)	C7—C8—C9	128.60 (9)
C9—N1—N2	117.07 (8)	O1—C8—C9	118.87 (8)
C9—N1—H1N1	117.6 (12)	O2—C9—N1	124.39 (9)
N2—N1—H1N1	125.2 (12)	O2—C9—C8	119.11 (9)
C10—N2—N1	115.70 (9)	N1—C9—C8	116.48 (8)
O1—C1—C2	125.76 (9)	N2—C10—C11	119.81 (9)
O1—C1—C6	110.21 (8)	N2—C10—H10A	120.1
C2—C1—C6	124.03 (9)	C11—C10—H10A	120.1
C1—C2—C3	115.97 (10)	C16—C11—C12	119.16 (10)
C1—C2—H2A	122.0	C16—C11—C10	121.81 (9)
C3—C2—H2A	122.0	C12—C11—C10	119.02 (9)
C2—C3—C4	121.76 (10)	C13—C12—C11	120.95 (10)
C2—C3—H3A	119.1	C13—C12—H12A	119.5
C4—C3—H3A	119.1	C11—C12—H12A	119.5
C5—C4—C3	121.07 (10)	C14—C13—C12	118.71 (10)
C5—C4—H4A	119.5	C14—C13—H13A	120.6
C3—C4—H4A	119.5	C12—C13—H13A	120.6
C4—C5—C6	118.35 (10)	C13—C14—C15	121.45 (10)
C4—C5—H5A	120.8	C13—C14—Cl1	119.90 (8)
C6—C5—H5A	120.8	C15—C14—Cl1	118.65 (9)
C5—C6—C1	118.81 (9)	C14—C15—C16	119.50 (10)
C5—C6—C7	135.37 (10)	C14—C15—H15A	120.2
C1—C6—C7	105.81 (9)	C16—C15—H15A	120.2
C8—C7—C6	105.94 (9)	C15—C16—C11	120.20 (10)
C8—C7—H7A	127.0	C15—C16—H16A	119.9
C6—C7—H7A	127.0	C11—C16—H16A	119.9
C7—C8—O1	112.48 (9)	H1W1—O1W—H2W1	107 (2)
			
C9—N1—N2—C10	175.60 (9)	N2—N1—C9—O2	0.48 (14)
C8—O1—C1—C2	−179.09 (10)	N2—N1—C9—C8	179.00 (8)
C8—O1—C1—C6	0.46 (11)	C7—C8—C9—O2	7.15 (16)
O1—C1—C2—C3	−179.16 (10)	O1—C8—C9—O2	−175.52 (9)
C6—C1—C2—C3	1.35 (16)	C7—C8—C9—N1	−171.45 (10)
C1—C2—C3—C4	−0.30 (16)	O1—C8—C9—N1	5.88 (13)
C2—C3—C4—C5	−0.69 (18)	N1—N2—C10—C11	−179.00 (8)
C3—C4—C5—C6	0.67 (17)	N2—C10—C11—C16	−2.28 (15)
C4—C5—C6—C1	0.32 (16)	N2—C10—C11—C12	176.82 (10)
C4—C5—C6—C7	−179.70 (12)	C16—C11—C12—C13	1.54 (16)
O1—C1—C6—C5	179.05 (9)	C10—C11—C12—C13	−177.59 (10)
C2—C1—C6—C5	−1.38 (16)	C11—C12—C13—C14	−1.15 (16)
O1—C1—C6—C7	−0.94 (11)	C12—C13—C14—C15	−0.46 (17)
C2—C1—C6—C7	178.62 (10)	C12—C13—C14—Cl1	179.76 (8)
C5—C6—C7—C8	−178.95 (12)	C13—C14—C15—C16	1.64 (17)
C1—C6—C7—C8	1.04 (11)	Cl1—C14—C15—C16	−178.57 (9)
C6—C7—C8—O1	−0.81 (12)	C14—C15—C16—C11	−1.23 (16)
C6—C7—C8—C9	176.66 (10)	C12—C11—C16—C15	−0.34 (16)
C1—O1—C8—C7	0.24 (11)	C10—C11—C16—C15	178.77 (10)
C1—O1—C8—C9	−177.51 (9)		

### Hydrogen-bond geometry (Å, º)

**Table:**

*D*—H···*A*	*D*—H	H···*A*	*D*···*A*	*D*—H···*A*
O1*W*—H1*W*1···O2	0.855 (19)	2.040 (19)	2.8815 (11)	168.1 (18)
O1*W*—H2*W*1···O2^i^	0.75 (2)	2.06 (2)	2.8045 (11)	173 (2)
N1—H1*N*1···O1*W*^ii^	0.909 (19)	1.952 (19)	2.8083 (12)	156.2 (18)
C2—H2*A*···O2^ii^	0.95	2.57	3.3710 (14)	142
C10—H10*A*···O1*W*^ii^	0.95	2.54	3.3067 (13)	138

Symmetry codes: (i) *x*, *y*−1, *z*; (ii) *x*, −*y*+1, *z*+1/2.
